# Supplementary material for: The interactome of intact mitochondria by cross-linking mass spectrometry provides evidence for coexisting respiratory supercomplexes
Source: Mol Cell Proteomics. 2017 Dec 8;17(2):216–32. doi: 10.1074/mcp.RA117.000470 (PMC5795388; doi:10.1074/mcp.RA117.000470)
Supplement: Supplemental Data [file supp_RA117.000470_133922_0_supp_23978_fzffwf.zip › spectra_annotation/mito_DR_spectra_annotation/390-1-5-1-18-1.pdf]

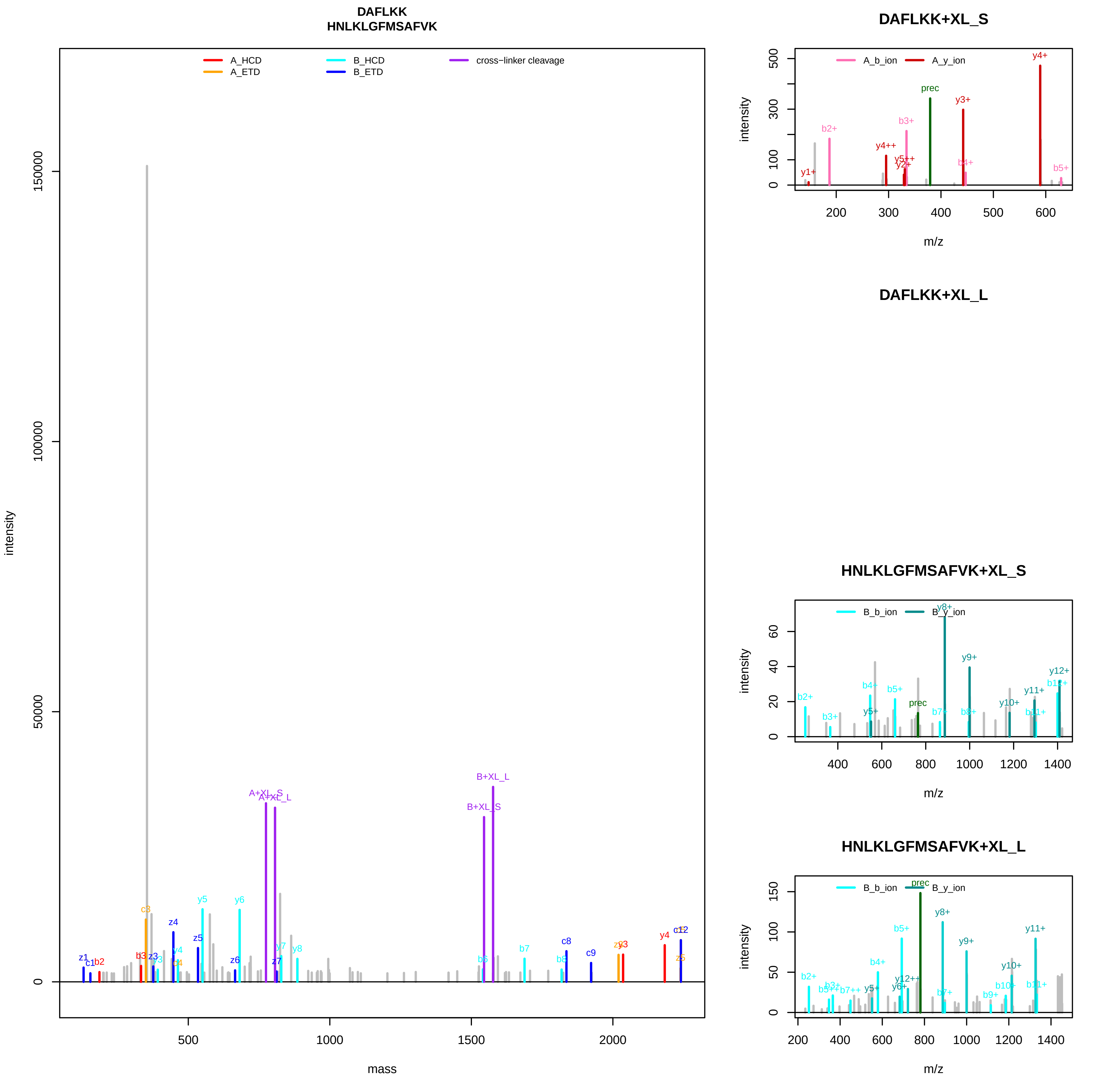
DAFLKK
HNLKLGFMSAFVK
A_HCD
A_ETD
B_HCD
B_ETD
cross−linker cleavage
intensity
mass
0
50000
100000
150000
500
1000
1500
2000
z1
c1
b2
b3
c3
z3
z4
y4
c4
z5
y5
z6
y6
A+XL_S
A+XL_L
z7
y7
y8
B+XL_S
B+XL_L
b6
b7
b8
c8
c9
y3
y4
c12
DAFLKK+XL_S
A_b_ion
A_y_ion
intensity
m/z
0
100
300
500
200
300
400
500
600
y1+
b2+
y4++
y5++
y2+
b3+
prec
y3+
b4+
y4+
b5+
DAFLKK+XL_L
HNLKLGFMSAFVK+XL_S
B_b_ion
B_y_ion
intensity
m/z
0
20
40
60
400
600
800
1000
1200
1400
b2+
b3+
b4+
y5+
b5+
prec
b7+
y8+
b8+
y9+
y10+
y11+
b11+
y12+
HNLKLGFMSAFVK+XL_L
B_b_ion
B_y_ion
intensity
m/z
0
50
100
150
200
400
600
800
1000
1200
1400
b2+
b3+
b5++
b7++
b4+
y5+
b5+
y6+
y12++
prec
y8+
b7+
y9+
b9+
y10+
b10+
y11+
b11+
